# Physicochemical Properties and Sensory Evaluation of Low-Sugar Collagen Jelly Using Fruit and Vegetable Powder

**DOI:** 10.3390/foods14193407

**Published:** 2025-10-01

**Authors:** Junho Yu, Seon-Joo Park, Hae-Jeung Lee

**Affiliations:** 1 Department of Food Science and Biotechnology, College of Bionanotechnology, Gachon University, Seongnam-si 13120, Republic of Korea; jumpjun9301@naver.com; 2 Department of Food and Nutrition, College of Bionanotechnology, Gachon University, Seongnam-si 13120, Republic of Korea; 3 Institute for Aging and Clinical Nutrition Research, Gachon University, Seongnam-si 13120, Republic of Korea

**Keywords:** collagen, jelly, powder, antioxidant, sensory evaluation

## Abstract

The collagen jelly market is expanding amidst rising consumer health consciousness. However, the high sugar and calorie contents of traditional collagen jellies make them unsuitable for patients with diabetes or obesity. The aim of this study is to develop a low-sugar collagen jelly using fruit and vegetable powder (apple, carrot, and tomato) and to identify the optimal manufacturing conditions by evaluating physicochemical and sensory properties. Texture profile analysis (TPA), proximate composition, color analysis, total flavonoid and polyphenol content, and antioxidant activity were evaluated in jellies containing 0–10% fruit and vegetable powder. Sensory evaluation on color, flavor, taste, texture, and overall preference was performed using a seven-point Likert scale. The antioxidant capacity of the jelly increased with the addition of the fruit and vegetable powder. Among the formulations evaluated, jelly containing 8% mixed powder achieved the highest preference score, highlighting its potential for consumer acceptance. This result can be attributed to the successful integration of a low-sugar base with antioxidant-rich powders, which offers both health benefits and strong consumer appeal.

## 1. Introduction

With growing consumer interest in health and beauty, the market for collagen jelly has also expanded [1]. Collagen is the main component of the skin, and the production of collagen in the skin decreases with age [2]. Collagen supplementation increases skin moisture retention, which reduces the appearance of wrinkles and fine lines [3,4,5]. Although collagen-containing jellies are widely consumed for perceived health and beauty benefits, they have high sugar and caloric contents that limit their suitability for consumers who need to manage blood glucose levels [6]. Excessive consumption of glucose and sucrose can rapidly elevate blood glucose levels and negatively affect insulin regulation in such populations [7]. While low-sugar food products are being developed with alternative sweeteners to replace sugar, concerns remain regarding the stability of these substitutes. Erythritol has been recognized as safe in both animal and human studies by the Joint WHO/FAO Expert Committee on Food Additives, as well as by food safety authorities in more than 60 countries [8]. 

Erythritol is absorbed in the small intestine and excreted in the urine without being metabolized in the body; therefore, it does not affect blood glucose levels, and it has a glycemic index of zero and no calorie value [9]. In addition, erythritol has been shown to efficiently scavenge free radicals and prevent endothelial dysfunction in diabetic mice [10]. Previous studies have indicated that sucrose-based jellies typically exhibit soluble sugar concentrations of approximately 21–23 °Brix [11], whereas those formulated with sugar substitutes demonstrate significantly reduced levels, around 3–4 °Brix [12]. This reduction in sugar content suggests the potential of formulations using alternative sweeteners such as erythritol to significantly lower glycemic response. Therefore, low-sugar collagen jellies formulated with erythritol may contribute to both blood glucose management and the promotion of skin health.

Fruit and vegetable powders offer several advantages, including ease of handling, improved transportability, and enhanced storage stability compared to concentrated fruit juice [13,14]. They also provide bioactive compounds such as polyphenols, flavonoids, and dietary fiber, which are associated with antioxidant activity and enhanced nutritional functionality [15,16]. Previous studies have indicated that the incorporation of fruit powders can improve the color, nutritional value, and visual quality of processed foods [17]. However, certain formulation challenges persist, including the maintenance of desirable gel texture, ensuring solubility and reconstitution, and preserving heat-sensitive compounds during processing [12,18,19]. Addressing these factors is crucial to determining the optimal concentration that balances nutritional functionality with consumer acceptability.

The aim of this study was to develop an antioxidant-rich, low-sugar collagen jelly using erythritol and an apple–tomato–carrot blended powder, and to evaluate the optimal concentration of this blend at which the jelly exhibits superior physicochemical, antioxidant, and sensory properties.

## 2. Materials and Methods

### 2.1. Materials and Reagents

Apple powder, carrot powder, tomato powder, glucomannan, locust bean gum, carrageenan, collagen, citric acid, sugar, erythritol, and salt were bought from an online shopping platform (Cite). Ethanol, Folin–Ciocalteu phenolic reagent (2 N), sodium nitrite, sodium hydroxide, aluminum chloride, quercetin, gallic acid, and sodium carbonate were purchased from Sigma-Aldrich (St. Louis, MO, USA).

### 2.2. Jelly Preparation

Collagen jelly was prepared in six formulations by incorporating hot air-dried apple, carrot, and tomato powders at an optimized ratio of 5:1:1 (*w*/*w*) and at concentrations of 0%, 2%, 4%, 6%, 8%, and 10% of the total mass. All fruit and vegetable powders were derived from whole fruits and vegetables, which were dried with their peels intact. 

Preliminary trials were conducted to assess sensory preference and color balance. The gelling agent was formulated using carrageenan, glucomannan, and locust bean gum, which were selected based on a preliminary study [20]. A composite gelling agent was prepared by combining glucomannan, locust bean gum, and carrageenan at a ratio of 1:0.8:0.2 (*w*/*w*) [21]. Furthermore, sucrose and erythritol were incorporated in equal quantities (8.5 g each per 100 g of jelly) to impart sweetness while reducing the overall sugar content, as determined in the conducted preliminary optimization trials. Citric acid (0.7 g) served as an acidulant, and a minor quantity of salt (0.2 g) was added to enhance flavor. Collagen was consistently maintained at 5 g across all formulations, while the water content was adjusted in accordance with the concentration of fruit and vegetable powder. Subsequently, fruit and vegetable powder, sucrose, erythritol, citric acid, salt, and collagen were blended with water and heated at 80 °C for 5 mins to dissolve. Glucomannan, locust bean gum, and carrageenan were added to the mixture and boiled to thicken for 5 min. The mixture was poured into jelly molds and stored at 4 °C for 24 h. The proportions of sucrose, erythritol, citric acid, salt, collagen, water, and the mixed gelling agent were based on the formulation ratios summarized in Table 1. 

### 2.3. Proximate Composition and Sugar Content Analysis

Crude protein content was determined via the Kjeldahl method, and crude fat content was determined using the acid hydrolysis method, at 60 °C with a Soxhlet extractor until a constant weight was obtained. The ash content was measured by burning the samples after pyrolysis at 550 °C until a constant weight was obtained [22]. Moisture content was determined by drying at 130 °C until a constant weight was achieved, using a moisture analyzer (MA160, Sartorius, Seongnam, Republic of Korea) [23]. The carbohydrate content was calculated by subtracting the crude fat, crude protein, ash, and moisture content from 100% [24]. For sugar content anaslysis, 1 g of the sample was weighed and mixed with 10 mL of distilled water (DW), followed by ultrasonic extraction for 1 h, and measured using a refractometer.

### 2.4. Chromaticity Determination

The color properties of the jelly were measured using a spectrophotometer (CM-5, Konica Minolta, Tokyo, Japan). Hunter color analysis, based on the anatomical features of the human eye and considers three types of photoreceptor cells and their sensitivity to blue, green, and red, was employed. Measurements of brightness (L, representing lightness from 0 [black] to 100 [white]), redness (a, indicating the red (+)-to-green (−) axis), and yellowness (b, indicating the yellow (+)-to-blue (−) axis) were averaged over three experimental replicates [25].

### 2.5. Texture Profile Analysis (TPA)

The texture of the jelly was measured using a texture analyzer (UTA™, Yeonjin Instruments, Seoul, Republic of Korea) equipped with a 5 mm diameter cylindrical plastic probe and controlled via the UTA Analysis Software program. Texture profile analysis (TPA) was conducted on the samples under the following conditions: a target deformation of 50% of the original sample height, a trigger force of 0.01 N, a pre-test speed of 60 mm/min, a test speed of 10 mm/min, and a post-test speed of 60 mm/min. The samples were sectioned into cubes measuring 1.5 × 1.5 × 1.5 cm, and each test was conducted at 25 °C with a rest interval of 5 s between the two compression cycles. TPA parameters included hardness, adhesiveness, cohesiveness, springiness, gumminess and chewiness. Each measurement was conducted in triplicate.

### 2.6. Antioxidant Capacity Analysis

Antioxidants from the jellies were extracted by adding 100 mg of jelly and 10 mL of 80% (*v*/*v*) ethanol to an Erlenmeyer flask. The extraction was performed in an ultrasonic bath at 25 °C for 60 min. Subsequently, the extract was centrifuged at 3000 rpm for 5 min. The resulting supernatant collected and filtered through a 0.45 μm syringe filter (SH25P04NL, Hyundai Micro, Seoul, Republic of Korea). The final filtrate was used for quantitative analysis [26].

### 2.7. Total Flavonoid Content Analysis

The total flavonoid content of the extracted sample was measured using a colorimetric assay method [27]. Briefly, 1 mL of the sample was mixed with 0.3 mL of 5% (*w*/*v*) NaNO_2_ and allowed to react for 5 min. Subsequently, 0.3 mL of 10% (*w*/*v*) AlCl_3_ was added, and the mixture was left to react for an additional 6 min. The reaction was then stopped by adding 2 mL of 1 N NaOH and 2.4 mL of DW. The absorbance was measured at 510 nm to quantify the flavonoid content. Quantification was conducted by constructing a calibration curve using quercetin as the standard substance. The unit of total flavonoids content was converted into mg quercetin equivalent (QE)/g dry weight.

### 2.8. Total Phenolic Content Analysis

The total phenolic content of the sample was determined using the Folin–Ciocalteu colorimetric method with slight modifications based on the procedure described by Kaur and Kapoor [28]. A 100 μL aliquot of the extract was diluted with 2.9 mL of distilled water. Then, 0.5 mL of Folin–Ciocalteu reagent was added to the diluted extract and reacted in the dark for 3 min. To terminate the reaction, 2 mL of 10% (*w*/*v*) Na_2_CO_3_ solution was added. The absorbance was then measured at 750 nm. A calibration curve was constructed using gallic acid as the standard, and the total polyphenol content was converted into mg of gallic acid equivalent (GAE) per gram of dry weight.

### 2.9. DPPH (2,2-diphenyl-1-picrylhydrazyl) Radical-Scavenging Activity

A 0.2 mM 2,2-diphenyl-1-picrylhydrazyl (DPPH) solution was prepared using ethanol. Subsequently, 100 mg of the extracted sample was mixed with 2.9 mL of the prepared 0.2 mM DPPH solution and allowed to react in darkness for 30 min. Following the reaction, the absorbance of the solution was measured at 517 nm using a spectrophotometer. The antioxidant capacity of the extracted samples was determined by constructing a standard calibration curve using vitamin C as the standard material, at concentrations of 50, 100, 150, and 200 mg/L. Results are expressed as milligrams of vitamin C equivalents (VCE) per 100 g [29].

### 2.10. ABTS (2,2ʹ-Azino-bis[3-ethylbenzothiazoline-6-sulfonic acid]) Radical-Scavenging Assay

The 2,2ʹ-azino-bis (3-ethylbenzothiazoline-6-sulfonic acid) (ABTS) solution was prepared by mixing 2.45 mM potassium persulfate and 7 mM ABTS solutions in a 1:1 ratio. The mixture was kept in the dark for 16 h to facilitate the radical formation, and then it was diluted with ethanol prior to use. For the antioxidant activity assay, 100 µL of the jelly extract was added to 3.9 mL of the diluted ABTS solution and incubated in the dark for 10 min. The absorbance of each sample was measured at 734 nm. Antioxidant capacity was calculated using a standard calibration curve (50, 100, 150, and 200 mg/L) prepared with vitamin C, and the results are expressed as mg VCE per 100 g of sample [30].

### 2.11. Sensory Evaluation

Sensory evaluation was conducted with a panel of 30 adults aged 19 years or older, who were recruited from Seoul and Gyeonggi Province. They assessed color, taste, order, texture, and overall preference on a seven-point Likert scale according to the powder concentrations (2%, 4%, 6%, 8%, and 10%). The rating scale ranged from 1 to 7, with 1 indicating ‘extremely disliked’ and 7 indicating ‘extremely liked’ [31,32]. 

The present study was approved by the Institutional Review Committee of Gachon University (IRB No. 1044396-202308-HR-158-01, approval date 29 August 2023), and informed consent was obtained from all participants.

### 2.12. Statistical Analysis

All measurements are expressed as the mean ± standard deviation based on results from three repeated experiments. Differences among three or more groups were analyzed using one-way ANOVA, and when significant difference was observed between groups, Duncan’s post hoc test was performed. Statistical analyses were conducted using SAS 9.4 (Statistical Analysis System, SAS Institute, Cary, NC, USA), and significance was determined at the *p* < 0.05 level.

## 3. Results and Discussion

### 3.1. Appearance and Color Analysis

**Figure 1:**
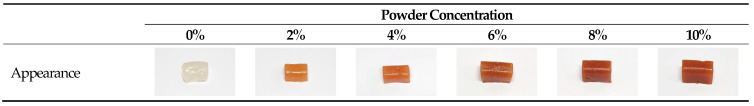
External appearance of collagen jellies formulated with different concentrations of fruit and vegetable powders.

The external appearance of the jelly according to the fruit and vegetable powder concentration is shown in Figure 1, and the corresponding color values are summarized in Table 2. The L value indicates brightness, while a and b values represent the red–green and yellow–blue color axes, respectively. As the concentration of fruit and vegetable powder increased, the L value decreased from 34.83 in the control to 23.60 at 10%, while the a value increased from −1.45 to 18.93 and the b value from 2.48 to 25.59. This trend can be attributed to the higher level of natural pigments, such as carotenoids from carrot and tomato and polyphenolic compounds from apple, which reduce brightness and increase redness and yellowness.

The present findings corroborate previous studies. Helyes et al. demonstrated that the ripening of tomatoes is associated with an accumulation of lycopene, resulting in decreased L values and increased a values [33]. Similarly, another study reported that the incorporation of grape pomace powder led to a reduction in L values while significantly enhancing a and b values in jelly products [34]. In our study, the jelly containing 10% fruit and vegetable powder exhibited the lowest L values and the highest a and b values, which is consistent with these observed trends.

### 3.2. Proximate Composition and Sugar Content Analysis

The proximate composition of the prepared jellies is shown in Table 3. The moisture content, crude protein, crude fat, ash, and total carbohydrate content were analyzed. As the proportion of fruit and vegetable powder increased, the moisture content decreased, while the crude protein, crude fat, and ash contents exhibited an increasing trend. This result is due to the natural composition of fruit and vegetable powder, which includes small amounts of crude protein, crude fat, and ash. Consequently, the total content of these components increases proportionally with the powder content. Furthermore, the observed decrease in moisture content can be attributed to the reduced water addition as the powder ratio increases. 

Although a decrease in moisture content is typically associated with lower water activity, which may potentially inhibit microbial growth [35], direct microbial stability was not assessed in this study. Conversely, the sugar content of the jelly increased with higher powder concentrations due to the intrinsic sugars present in apple, carrot, and tomato powders. The °Brix value rose from 2.43 in the control to 3.63 with a 10% powder addition. Previous studies on carrot jellies formulated with sugar substitutes have reported °Brix values ranging from 3.1 to 3.9 [12], indicating that our findings are consistent with other low-sugar formulations. In contrast, citrus jellies sweetened with sucrose typically exhibit °Brix values between 21 and 23 [11], underscoring that our collagen jelly can still be classified as a low-sugar product despite the presence of natural sugars.

### 3.3. Texture Profile Analysis

The textural properties of jellies prepared according to the fruit and vegetable powder concentrations are shown in Table 4. Hardness is used to measure how hard the food is at the compression stage [36]. The hardness increased significantly as the concentration of the powder increased. This observation suggests that the jelly structure became more rigid due to the reduction in moisture content. Adhesiveness is considered an important property of jelly because if this is too high, the jelly could stick to the teeth, palate, and tongue [37]. In this study, adhesiveness did not exhibit significant variation up to 6%; however, a significant increase was observed at 8% and 10%. Cohesiveness, defined as the ratio of the area under the second compression curve to that under the first compression curve (second bite/first bite), increased up to 4% but subsequently declined, with a noticeable reduction observed at higher concentrations (8% and 10%). Initially, the incorporation of powder into the jelly network served to enhance its cohesiveness by reinforcing the structure. However, when the concentration surpassed 8%, the excessive amount of powder led to a reduction in the availability of free water, thereby disrupting the uniform formation of the gel matrix. This phenomenon is consistent with observations in dense hydrocolloid systems, where excessive loading can compromise the homogeneity of the network [38,39]. Springiness, calculated as the ratio of the recovered height after compression to the original sample height, increased gradually up to 8% (highest value), but decreased at 10%. This indicates that a moderate quantity of powder contributes to maintaining the elasticity of the jelly, with the 8% concentration offering the most optimal balance. At a concentration of 10%, the observed reduction in elasticity likely results from a structural imbalance induced by an excessive load on the gel network [40]. Gumminess, defined as the product of hardness and cohesiveness, increased gradually, with the highest value at 8%, while the value at 10% did not differ significantly from 8%. This implies that up to 8%, the jelly maintained its viscoelastic properties, but at 10%, excessive powder weakened the internal structure, resulting in reduced gumminess. Chewiness, calculated as gumminess × springiness, increased consistently up to 8% and then decreased at 10%.

TPA revealed that jellies with lower concentrations of fruit and vegetable powder exhibited relatively low hardness, whereas those with higher powder concentrations showed increased hardness, resulting in a firmer and more brittle texture. As the powder concentration increased, the moisture content decreased, leading to a firmer and more fragile texture. Notably, cohesiveness, springiness, gumminess, and chewiness all exhibited peak values at mid-range concentrations before decreasing. The findings align with the sensory evaluation, wherein the 8% powder jelly was most favored. This suggests that moderate levels of powder yielded a texture that was firm, cohesive, and sufficiently elastic to meet consumer acceptance. It is particularly crucial to avoid excessive hardness or fragility, thereby reinforcing the appropriateness of the 8% formulation in the context of diabetic-friendly products. Achieving an optimal balance between sugar content and texture is crucial for products tailored to individuals with diabetes. These individuals necessitate not only a reduction in sugar levels but also a texture that ensures both comfort and palatability. Products that are excessively hard or brittle may discourage consistent consumption, whereas a texture that is moderate and agreeable can facilitate the acceptance of healthier, low-sugar alternatives.

### 3.4. Total Flavonoid and Polyphenol Content

The data pertaining to the total flavonoid and polyphenol contents in jellies, influenced by varying concentrations of fruit and vegetable powder, are presented in Table 5. Both total flavonoid content (TFC) and total polyphenol content (TPC) exhibited a consistent and significant increase with the augmentation of powder concentration. Specifically, the TFC increased from 1.58 mg QE/100 g at 0% to 12.80 mg QE/100 g at 8% and 14.99 mg QE/100 g at 10%. Similarly, the TPC rose from 12.72 mg GAE/100 g at 0% to 34.31 mg GAE/100 g at 8% and 39.99 mg GAE/100 g at 10%. The significant increases can be attributed to the flavonoid and phenolic compounds inherently present in the fruit and vegetable powder. The observed increase in TFC and TPC directly correlates with the enhanced antioxidant capacity of the jelly, as flavonoids and polyphenols are recognized for their role as radical scavengers and their ability to mitigate oxidative stress. Consequently, the incorporation of fruit and vegetable powders not only augments the phytochemical profile but also underscores the potential of this collagen jelly as a functional food with significant antioxidant properties.

### 3.5. DPPH and ABTS Radical-Scavenging Activity

The antioxidant properties of jellies, as evaluated using DPPH and ABTS radical-scavenging assays, are presented in Table 6. Both assays revealed a significant and progressive increase in radical-scavenging capacity with the augmented concentration of fruit and vegetable powder. Specifically, the DPPH activity increased from 25.68 mg VCE/100 g at 0% to 78.57 mg VCE/100 g at 8% and 90.67 mg VCE/100 g at 10%. Similarly, the ABTS activity rose from 12.68 mg VCE/100 g at 0% to 42.26 mg VCE/100 g at 8% and 51.72 mg VCE/100 g at 10%. These results confirm that the incorporation of fruit and vegetable powder is closely associated with enhanced antioxidant activity, attributable to phytochemicals such as polyphenols and flavonoids naturally present in apples, carrots, and tomatoes [41,42,43].

Previous research has indicated that increasing tomato concentration in jellies enhances both DPPH and ABTS radical-scavenging activities [44]. Moreover, research indicates that dried apple peel demonstrates greater antioxidant activity compared to peeled apples. This finding suggests that incorporating fruit powders containing peels may further augment antioxidant potential [45]. It is important to note that in comparison to fresh fruit or juice, which may experience losses of vitamin C and other bioactive compounds during heating and processing [46], the utilization of fruit and vegetable powders could potentially offer enhanced antioxidant stability, as indicated by previous studies [47,48,49]. Nevertheless, this potential benefit necessitates further validation, as stability was not directly assessed in this study.

### 3.6. Sensory Evaluation

The findings of the sensory evaluation are detailed in Table 7. The evaluation employed a seven-point Likert scale to measure the color, odor, taste, texture, and overall preference of jellies with varying concentrations of fruit and vegetable powder. Regarding color, the lowest score was 3.47 at 2%, whereas the highest score was 4.87 at 6%; for odor, the lowest score was 3.17 at 2%, and the highest score was 4.30 at 8%. Taste scores ranged from 3.67 at 2% to 4.63 at 8%, with the latter being the most preferred. Regarding texture, scores ranged from 3.23 at 4% to 3.93 at 6%, with no statistically significant differences observed among the groups. This is consistent with the mechanical texture profile analysis, which indicated objective differences not entirely captured by subjective sensory perception. Overall preference scores ranged from 3.17 at a 2% concentration to 4.30 at an 8% concentration, indicating that the latter was the most favorable formulation. Notably, the 8% powder level yielded the highest scores for odor, taste, and overall preference, which corresponded with the enhanced antioxidant capacity and improved phytochemical content observed in prior analyses. These findings suggest that the 8% formulation offers a balance between desirable sensory quality and functional benefits. Although the panel size of 30 participants was considered appropriate, it may not be entirely sufficient to ensure their generalizability.

## 4. Conclusions

This study developed a low-sugar collagen jelly by incorporating erythritol and fruit and vegetable powder. Among the formulations tested, the jelly containing 8% powder exhibited the most favorable balance, demonstrating enhanced antioxidant activity and high consumer acceptability. The observed decrease in brightness (L) and the increase in redness (a) and yellowness (b) confirmed that the color characteristics were influenced by the powder concentration. Textural analysis revealed that hardness and adhesiveness increased with higher powder concentration, while cohesiveness, springiness, gumminess, and chewiness peaked at intermediate levels and declined at 10%. These findings suggest that a moderate addition of powder improves structural and sensory properties, whereas excessive addition may impair them. The use of erythritol, a safe alternative sweetener with a zero glycemic index and no calories, underscores a strength of this formulation by enabling sugar reduction without compromising quality. However, this study has some limitations that should be considered. First, microbial stability was not evaluated, so the potential impact of moisture reduction on shelf life remains speculative. Second, the sensory panel consisted of only 30 participants, which limits the generalizability of the results. Third, this study did not compare the consumer preference and antioxidant capacity of jellies prepared with concentrated juice and those prepared with fruit and vegetable powder. Fourth, while the selected fruit and vegetable powders are acknowledged for their substantial dietary fiber content, an analysis of dietary fiber was not performed due to analytical limitations and scope constraints. Future research should incorporate microbial stability testing, larger sensory panels, and dietary fiber analysis to provide a more comprehensive understanding of the nutritional value and shelf life of these formulations. Despite these limitations, this research provides important insights into the formulation of functional low-sugar collagen jelly. The incorporation of 8% fruit and vegetable powder represents a viable strategy to enhance antioxidant potential and consumer preference, offering a nutritionally improved alternative to conventional high-sugar collagen jellies, particularly suitable for individuals requiring blood sugar control, such as patients with diabetes, by utilizing erythritol as a safe sugar substitute.

## Figures and Tables

**Table 1:** Compositional ratios of ingredients in six types of collagen jelly.

Ingredients	Powder Concentration					
	0%	2%	4%	6%	8%	10%
Fruit and vegetable powder (g) *	0	2	4	6	8	10
Erythritol (g)	8.5	8.5	8.5	8.5	8.5	8.5
Sugar (g)	8.5	8.5	8.5	8.5	8.5	8.5
Citric acid (g)	0.7	0.7	0.7	0.7	0.7	0.7
Salt (g)	0.2	0.2	0.2	0.2	0.2	0.2
Mixed gellant (g) **	2	2	2	2	2	2
Collagen (g)	5	5	5	5	5	5
Water (g)	75.1	73.1	71.1	69.1	67.1	65.2

* Powder: apple, carrot, and tomato powder were mixed in a ratio of 5:1:1. ** Mixed gellant: Glucomannan, locust bean gum, and carrageenan were mixed in a ratio of 1:0.8:0.2.

**Table 2:** Hunter color analysis of collagen jellies according to fruit and vegetable powder concentration.

	Powder Concentration					
	0%	2%	4%	6%	8%	10%
Lightness (L)	34.83 ± 0.04 ^a^	31.17 ± 0.07 ^b^	28.33 ± 0.05 ^c^	27.39 ± 0.18 ^d^	25.27 ± 0.12 ^e^	23.60 ± 0.19 ^f^
Redness (a)	−1.45 ± 0.01 ^e^	10.82 ± 0.04 ^d^	14.90 ± 0.07 ^c^	17.28 ± 0.17 ^b^	17.49 ± 0.11 ^b^	18.93 ± 0.23 ^a^
Yellowness (b)	2.48 ± 0.02 ^e^	15.07 ± 0.03 ^e^	20.84 ± 0.02 ^d^	23.08 ± 0.20 ^c^	24.29 ± 0.07 ^b^	25.59 ± 0.10 ^a^

All values are expressed as mean ± standard deviation of triplicate determinations. ^a–f^: Different superscripts within the same row indicate significant differences (*p* < 0.05) via Duncan’s multiple range test.

**Table 3:** Proximate composition and sugar content of collagen jelly according to fruit and vegetable powder concentration (%).

	Powder Concentration					
	0%	2%	4%	6%	8%	10%
Moisture (%)	50.89 ± 0.50 ^a^	47.80 ± 3.72 ^ab^	45.80 ± 2.78 ^bc^	43.81 ± 1.05 ^cd^	41.52 ± 0.97 ^de^	39.70 ± 1.20 ^e^
Crude protein (%)	6.28 ± 0.20 ^e^	6.63 ± 0.20 ^de^	6.86 ± 0.34 ^cd^	7.08 ± 0.20 ^bc^	7.43 ± 0.20 ^ab^	7.66 ± 0.20 ^a^
Crude fat (%)	0.31 ± 0.09 ^d^	0.43 ± 0.03 ^de^	0.57 ± 0.06 ^cd^	0.70 ± 0.12 ^bc^	0.90 ± 0.13 ^ab^	1.09 ± 0.21 ^a^
Ash (%)	0.40 ± 0.14 ^f^	0.72 ± 0.03 ^e^	0.93 ± 0.07 ^d^	1.21 ± 0.08 ^c^	1.47 ± 0.10 ^b^	1.73 ± 0.18 ^a^
Total carbohydrates (%)	42.11 ± 10.46 ^d^	44.42 ± 3.90 ^cd^	45.84 ± 2.61 ^bcd^	47.19 ± 1.03 ^abc^	48.68 ± 0.77 ^ab^	49.83 ± 1.10 ^a^
Sugar (°Brix)	2.43 ± 0.06 ^f^	2.67 ± 0.06 ^e^	2.93 ± 0.06 ^d^	3.17 ± 0.06^c^	3.43 ± 0.06^b^	3.63 ± 0.06 ^a^

All values are expressed as mean ± standard deviation of triplicate determinations. ^a–f^: Different superscripts within the same row indicate significant differences (*p* < 0.05) via Duncan’s multiple range test.

**Table 4:** Mechanical parameters of collagen jelly according to fruit and vegetable powder concentration (%) via TPA *.

	Powder Concentration					
	0%	2%	4%	6%	8%	10%
Hardness (N)	1.32 ± 0.15 ^d^	1.50 ± 0.07 ^cd^	1.61 ± 0.01 ^cd^	1.77 ± 0.11 ^c^	2.51 ± 0.36 ^b^	3.39 ± 0.27 ^a^
Adhesiveness (N·s)	0.04 ± 0.03 ^c^	0.06 ± 0.04 ^c^	0.06 ± 0.00 ^c^	0.07 ± 0.01 ^c^	0.19 ± 0.07 ^b^	0.26 ± 0.03 ^a^
Cohesiveness (-)	0.65 ± 0.03 ^a^	0.66 ± 0.01 ^a^	0.68 ± 0.03 ^a^	0.64 ± 0.02 ^a^	0.53 ± 0.04 ^b^	0.34 ± 0.03 ^c^
Springiness (-)	0.52 ± 0.08 ^b^	0.59 ± 0.06 ^ab^	0.68 ± 0.05 ^a^	0.69 ± 0.05 ^a^	0.72 ± 0.05 ^a^	0.60 ± 0.09 ^ab^
Gumminess (N)	0.86 ± 0.13 ^c^	1.00 ± 0.04 ^bc^	1.09 ± 0.04 ^ab^	1.13 ± 0.09 ^ab^	1.33 ± 0.22 ^a^	1.17 ± 0.17 ^ab^
Chewiness (N)	0.45 ± 0.08 ^d^	0.59 ± 0.07 ^cd^	0.74 ± 0.08 ^bc^	0.79 ± 0.11 ^b^	0.95 ± 0.10 ^a^	0.70 ± 0.07 ^bc^

All values are expressed as mean ± standard deviation of triplicate determinations. ^a–d^: Different superscripts within the same row indicate significant differences (*p* < 0.05) via Duncan’s multiple range test. * Texture profile analysis.

**Table 5:** Total polyphenol and flavonoid contents of collagen jelly according to fruit and vegetable powder concentration (%).

	Powder Concentration					
	0%	2%	4%	6%	8%	10%
Total flavonoid contents (mg QE/g)	1.58 ± 0.07 ^f^	5.13 ± 0.29 ^e^	8.57 ± 0.34 ^d^	10.74 ± 0.28 ^c^	12.80 ± 0.22 ^b^	14.99 ± 0.07 ^a^
Total polyphenol contents (mg GAE/g)	12.72 ± 0.12 ^f^	16.91 ± 0.12 ^e^	22.48 ± 0.10 ^d^	28.32 ± 0.05 ^c^	34.31 ± 0.10 ^b^	39.99 ± 0.20 ^a^

All values are expressed as mean ± standard deviation of triplicate determinations. ^a–f^: Different superscripts within the same row indicate significant differences (*p* < 0.05) via Duncan’s multiple range test.

**Table 6:** DPPH and ABTS radical-scavenging activity of collagen jelly according to fruit and vegetable powder concentration (%).

	Powder Concentration					
	0%	2%	4%	6%	8%	10%
DPPH (mg VCE/100g)	25.68 ± 0.71 ^f^	36.48 ± 0.44 ^e^	50.39 ± 0.66 ^d^	63.40 ± 0.48 ^c^	78.57 ± 0.28 ^b^	90.67 ± 0.24 ^a^
ABTS (mg VCE/100g)	12.68 ± 0.34 ^f^	19.37 ± 0.22 ^e^	25.81 ± 0.30 ^d^	34.10 ± 0.05 ^c^	42.26 ± 0.25 ^b^	51.72 ± 0.25 ^a^

All values are expressed as mean ± standard deviation of triplicate determinations. ^a–f^: Different superscripts within the same row indicate significant differences (*p* < 0.05) via Duncan’s multiple range test.

**Table 7:** Sensory evaluation of collagen jelly according to fruit and vegetable powder concentration (%).

	Powder Concentration				
	2%	4%	6%	8%	10%
Color	3.47 ± 1.14 ^c^	4.50 ± 1.31 ^ab^	4.87 ± 0.97 ^a^	4.67 ± 1.03 ^ab^	4.13 ± 1.20 ^b^
Odor	3.17 ± 1.18 ^c^	3.40 ± 1.10 ^bc^	3.93 ± 1.05 ^ab^	4.30 ± 1.39 ^a^	3.80 ± 1.21 ^abc^
Taste	3.67 ± 1.30 ^b^	4.13 ± 1.11 ^ab^	4.13 ± 1.33 ^ab^	4.63 ± 1.10 ^a^	4.03 ± 1.47 ^ab^
Texture	3.40 ± 1.28 ^ns^	3.23 ± 1.41	3.93 ± 1.17	3.87 ± 1.14	3.40 ± 1.45
Overall preference	3.17 ± 1.21 ^b^	3.77 ± 1.30 ^ab^	4.13 ± 1.14 ^a^	4.30 ± 1.09 ^a^	3.70 ± 1.42 ^ab^

All values are expressed as mean ± standard deviation of triplicate determinations. ^a–c^: Different superscripts within the same row indicate significant differences (*p* < 0.05) via Duncan’s multiple range test. ^ns^ not significant.

## Data Availability

The original contributions presented in this study are included in the article. Further inquiries can be directed to the corresponding authors.
